# Quality appraisal and descriptive analysis of clinical practice guidelines for self-managed non-pharmacological interventions of cardiovascular diseases: a systematic review

**DOI:** 10.1186/s12967-024-04959-5

**Published:** 2024-02-29

**Authors:** Yun Qian, Jing-Yu (Benjamin) Tan, Tao Wang, Daniel Bressington, Hong-Juan Zhou, Meng-Yuan Li, Xian-Liang Liu

**Affiliations:** 1 Faculty of Health, Charles Darwin University, Casuarina, Australia; 2 School of Nursing and Midwifery, University of Southern Queensland, Ipswich, QLD Australia; 3 School of Nursing, Putian University, Putian, Fujian China; 4 School of Nursing and Health Studies, Hong Kong Metropolitan University, Homantin, Kowloon, Hong Kong, China; 5 Centre for Health Research, University of Southern Queensland, Springfield, QLD Australia; 6 Maroondah Hospital, Eastern Health, Melbourne, Australia

**Keywords:** Cardiovascular disease, Systematic review, Clinical practice guideline, Self-management, Non-pharmacological interventions

## Abstract

**Background:**

Cardiovascular diseases (CVDs) are the leading cause of death around the world. Most CVDs-related death can be prevented by the optimal management of risk factors such as unhealthy diet and physical inactivity. Clinical practice guidelines (CPGs) for CVDs, provide some evidence-based recommendations which help healthcare professionals to achieve the best care for patients with CVDs. This systematic review aims to appraise the methodological quality of CPGs systematically and summarize the recommendations of self-managed non-pharmacological interventions for the prevention and management of CVDs provided by the selected guidelines.

**Methods:**

A comprehensive electronic literature search was conducted via six databases (PubMed, Medline, The Cochrane Library, Embase, CINAHL, and Web of Science), seven professional heart association websites, and nine guideline repositories. The Appraisal of Guidelines, Research and Evaluation II (AGREE II) instrument was adopted to critically appraise the methodological quality of the selected guidelines. Content analysis was used to summarise recommended self-managed non-pharmacological interventions for CVDs.

**Results:**

Twenty-three CPGs regarding different CVDs were included, in which four guidelines of CVDs, three for coronary heart diseases, seven for heart failure, two for atrial fibrillation, three for stroke, three for peripheral arterial disease, and one for hypertrophic cardiomyopathy. Twenty CPGs were appraised as high quality, and three CPGs as moderate quality. All twenty-three CPGs were recommended for use with or without modification. The domain of “Editorial Independence” had the highest standardized percentage (93.47%), whereas the domain of “Applicability” had the lowest mean domain score of 75.41%. The content analysis findings summarised some common self-managed non-pharmacological interventions, which include healthy diet, physical activity, smoking cessation, alcohol control, and weight management. Healthy diet and physical acidity are the most common and agreed on self-managed interventions for patients with CVDs. There are some inconsistencies identified in the details of recommended interventions, the intervention itself, the grade of recommendation, and the supported level of evidence.

**Conclusion:**

The majority of the summarized non-pharmacological interventions were strongly recommended with moderate to high-quality levels of evidence. Healthcare professionals and researchers can adopt the results of this review to design self-managed non-pharmacological interventions for patients with CVDs.

## Introduction

Cardiovascular diseases (CVDs) are a group of diseases affecting the heart and blood vessels, which include coronary heart disease, cerebrovascular disease, peripheral arterial disease and other conditions [1]. There were about 523 million people diagnosed with CVDs and 18.6 million CVD deaths (representing 32% of all global deaths) in 2019, approximately 19.1 million CVD deaths in 2020, which made CVDs the major contributor to disability and the leading cause of mortality worldwide [2, 3]. CVDs have a major impact on an individual’s personal health and quality of life and cause economic burdens for the individual, family, and the whole society. Despite the high morbidity and mortality rate, about 90% CVDs are preventable, making CVDs prevention one of the most deserving research topics [4].CVDs are caused by multiple factors, including invariable factors (e.g. genetic heritage, age, gender) and variable factors (e.g. unhealthy diet and harmful use of alcohol, physical inactivity, obesity, tobacco use) [1, 5]. According to the Australian Burden of Disease Study 2018, about 68% of CVD burden is related to modifiable risk factors [6].

Patients with CVD face significant challenges in managing the disease and maintaining a healthy lifestyle as CVDs are chronic conditions that require long-term and continuous care[7]. CVDs have strong association with behavioural factors (e.g. lifestyle, including eating habits, smoking and physical activity) and psychosocial factors (e.g. work or family stress, anxiety) [5]. Secondary prevention of CVDs focus on early diagnosis and treatment to avoid life threatening situations and long term impairments from CVDs [4, 8]. Secondary prevention is essential in reducing the CVD burden because it targets modifiable risk factors and encourages lifestyle changes, leading to better health outcomes and improved quality of life for patients [5, 9]. Moreover, the cost of secondary prevention is significantly lower than tertiary prevention, which often involves invasive procedures and hospitalizations [4]. Secondary prevention of CVDs includes medical treatment, risk factors modification, psychosocial care, education and support for self-management [10]. Compared to tertiary prevention which requires major procedures causing patient discomfort and disruption of daily activities, secondary prevention emphasizes less intense treatment [4]. Therefore, raising awareness of secondary prevention will have a positive impact on both individual lives and the macroeconomic level [2, 4]. By empowering patients to take control of their health and make informed decisions about their care, secondary prevention can play a vital role in reducing the burden of CVDs on individuals, families, and society as a whole [2, 11, 12].

Self-management can be defined as “the individual’s ability to manage the symptoms, treatment, physical and psychological consequences, and lifestyle changes inherent in living with a chronic condition” [13]. Self-management has been widely accepted as an effective way to support patients to achieve better quality of life while living with chronic conditions [14, 15]. One systematic review and meta-analysis including 24 randomized controlled trials (RCTs) with 9634 participants have demonstrated that self-management programs can improve quality of life and reduce readmissions of patients with heart failure [16]. 2021 ESC guideline for heart failure (HF) strongly recommended self-management to reduce HF hospitalization and mortality based on high level of evidence (LOE A) [17]. There are a few guidelines of CVDs which recommended self-management interventions for patients with CVDs focusing on risk factors modification, such as healthy diet, smoking cessation and weight management [18–22]. Since the treatment of CVDs include the specific medication use and adherence to non-pharmacological interventions, non-pharmacological interventions can be self-managed and are important to the successful management of CVDs [23], We defined self-managed non-pharmacological interventions as the interventions for both physical and psychosocial aspects of chronic conditions that patients can manage at home independently or with assistance of family/carer, but with minimal support of clinicians [13, 24].

Clinical practice guidelines are systematically developed statements based on best available evidence, designed to assist decision making and optimise patient care for specific circumstances [25]. Recent research have shown that the implementation of CPGs and good adherence to CPGs can improve the care process, clinical and public outcomes [26–28]. Optimal adherence to the lifestyle measures and medical therapy as recommended by guidelines is the key to successful secondary prevention of CVDs [29].However, it has been found that the adherence to cardiovascular guidelines was unsatisfactory, due to some barriers in implementation, including intrinsic factors such as clinician’s understanding and attitudes, as well as extrinsic factors such as patient’s preferences, guideline clarity and complexity, and environmental constraints [28].

The quality of CPG including the recommendations’ applicability, variability, accessibility and complexity may impact the implementation of CPG [28]. To improve the quality of CPGs, many methodologies and principles have been established, however, the adherence of the methodologies and development strategies remain uncertain during guideline development [30]. There are many CPGs regarding CVDs which include self-managed non-pharmacological interventions. Due to different CPGs produced by different organizations or countries, the methodological quality of the CPGs, the recommendations of self-managed non-pharmacological interventions and the level of evidence underpinning the recommendations varies across guidelines [17–21, 31]. Healthcare professionals may find it quite confusing and frustrated to provide best care for patients when the quality of the CPG is uncertain and the recommendations are inconsistent [32].

To our best knowledge, there is no recent review which has appraised the quality of CPGs for self-managed non-pharmacological interventions of patients with CVDs. This review is necessary because self-managed non-pharmacological interventions can play a crucial role in improving patients' quality of life and overall health outcomes. By examining these guidelines, we can identify inconsistencies and gaps in recommendations, ultimately improving the implementation and effectiveness of CVD management. Therefore, the objectives of this systematic review were: (1) to systematically appraise the methodological quality of included CPGs for the management of CVDs by using AGREE II; (2) to summarize and analyse the recommendations of self-managed non-pharmacological interventions for patients with CVDs, including the degree of recommendation and level of evidence.

## Methods

The systematic review protocol is registered at INPLASY (INPLASY 202250030). Standard systematic review methodology has been used to guide this review [33] and Preferred Reporting Items for Systematic Reviews and Meta-Analyses Protocols (PRISMA-P) checklist has been used to guide the report of this systematic review [34].

### Search strategies

A comprehensive literature search was conducted to identify relevant CPGs published from Jan 2017 to May 2022 and the search language was limited to English only. PubMed, CINAHL, Medline, The Cochrane Library, Embase, and Web of Science were searched. Seven professional association websites were searched, which included World Heart Federation, The American Heart Association/American Stroke Association, National Heart Foundation of Australia, Cardiac Society of Australia and New Zealand (CSANZ), British Heart Foundation, Heart and Stroke Foundation of Canada, and European Society of Cardiology. The websites of guideline developing organizations were searched as well, including the Australian Clinical Practice Guidelines Portal, CPG Infobase, Canadian Medical Association, the Guideline International Network, the Scottish Intercollegiate Guidelines Network, the National Guideline Clearinghouse (NGC), National Institute for Health and Care Excellence (NICE), the New Zealand Guidelines Group, National Health and Medical Research Council (NHMRC), and the Turning Research Into Practice (TRIP) database. MeSH terms including cardiovascular diseases, heart diseases, guidelines as topic, guideline, consensus, self care, self-management, healthy lifestyle, diet, exercise, smoking cessation and weight loss. Suitable search strategies including key terms and keywords were tailored for different database to identify possible guidelines. The search method in PubMed is presented as a representative search strategy (Table 1).

**Table 1:** Searching strategy example (PubMed)

	Search strategies	Number of records
#1	((((((((Cardiovascular diseases [MeSH Terms]) OR (Heart Diseases[MeSH Terms])) OR (Cardiovascular[Title/Abstract])) OR (Atherosclerotic Cardiovascular[Title/Abstract])) OR (Coronary Artery[Title/Abstract])) OR (Coronary Heart[Title/Abstract])) OR (Ischaemic Heart[Title/Abstract])) OR (Stroke[Title/Abstract])) OR (cardiac[Title/Abstract])	3,216,964
#2	((((((((((Guidelines as Topic[MeSH Terms]) OR (guideline[MeSH Terms])) OR (Consensus[MeSH Terms])) OR (Guidelin*[Title/Abstract])) OR (Best Practice*[Title/Abstract])) OR (Recommendation*[Title/Abstract])) OR (Consensus*[Title/Abstract])) OR (Expert Opinion*[Title/Abstract])) OR (Pathway*[Title/Abstract])) OR (evidence-based[Title/Abstract])) OR (evidence based[Title/Abstract])	2,310,627
#3	(((((((((((((((((((((((Self care[MeSH Terms]) OR (self-management[MeSH Terms])) OR (healthy lifestyle[MeSH Terms])) OR (diet, healthy[MeSH Terms])) OR (exercise[MeSH Terms])) OR (smoking cessation[MeSH Terms])) OR (weight loss[MeSH Terms])) OR (Non-pharmacological[Title/Abstract])) OR (lifestyle[Title/Abstract])) OR (self-manage*[Title/Abstract])) OR (self manage*[Title/Abstract])) OR (self-monitoring[Title/Abstract])) OR (self care*[Title/Abstract])) OR (self regulat*[Title/Abstract])) OR (self help*[Title/Abstract])) OR (self efficacy[Title/Abstract])) OR (self direct*[Title/Abstract])) OR (self maintain*[Title/Abstract])) OR (self monitor*[Title/Abstract])) OR (diet[Title/Abstract])) OR (physical activit*[Title/Abstract])) OR (smoking[Title/Abstract])) OR (weight control[Title/Abstract])) OR (weight reduction[Title/Abstract])	1,138,186
#4	#1 AND #2 AND #3	22,546
#5	Filter: Full text, in the last 5 years	8217
#6	Filter: Full text, in the last 5 years, Humans	5955
#7	Filter: Full text, in the last 5 years, Humans, English	5791
#8	Filter: Full text, in the last 5 years, Humans, English, Review	1753

PubMed (search done 05/2022)

### Eligibility criteria

Guidelines with full text versions were included if they met the eligibility criteria: (1) Designed for adult patients with CVDs (defined as a group of heart and blood vessels disorders, including but not limited to coronary artery disease, stroke, heart failure, valvular heart disease, and peripheral artery disease [1]; (2) include any type of self-managed non-pharmacological interventions, such as exercise, diet control, weight control, behavioural intervention for alcohol and cigarette uses; (3) designed for use by healthcare professionals; (4) published in English; (5) published from Jan 2017 to May 2022, because CPGs require regular evaluation and updates within the timeframe of 2–5 years to make sure all the recommendations are valid due to changes in clinical evidence and healthcare resources [35, 36]; (6) the latest version if there are successive editions; (7) with clear criteria for level of evidence and grade of recommendation; (8) source of evidence generally based on empirical research evidence.

### Study selection and data extraction

**Fig. 1:**
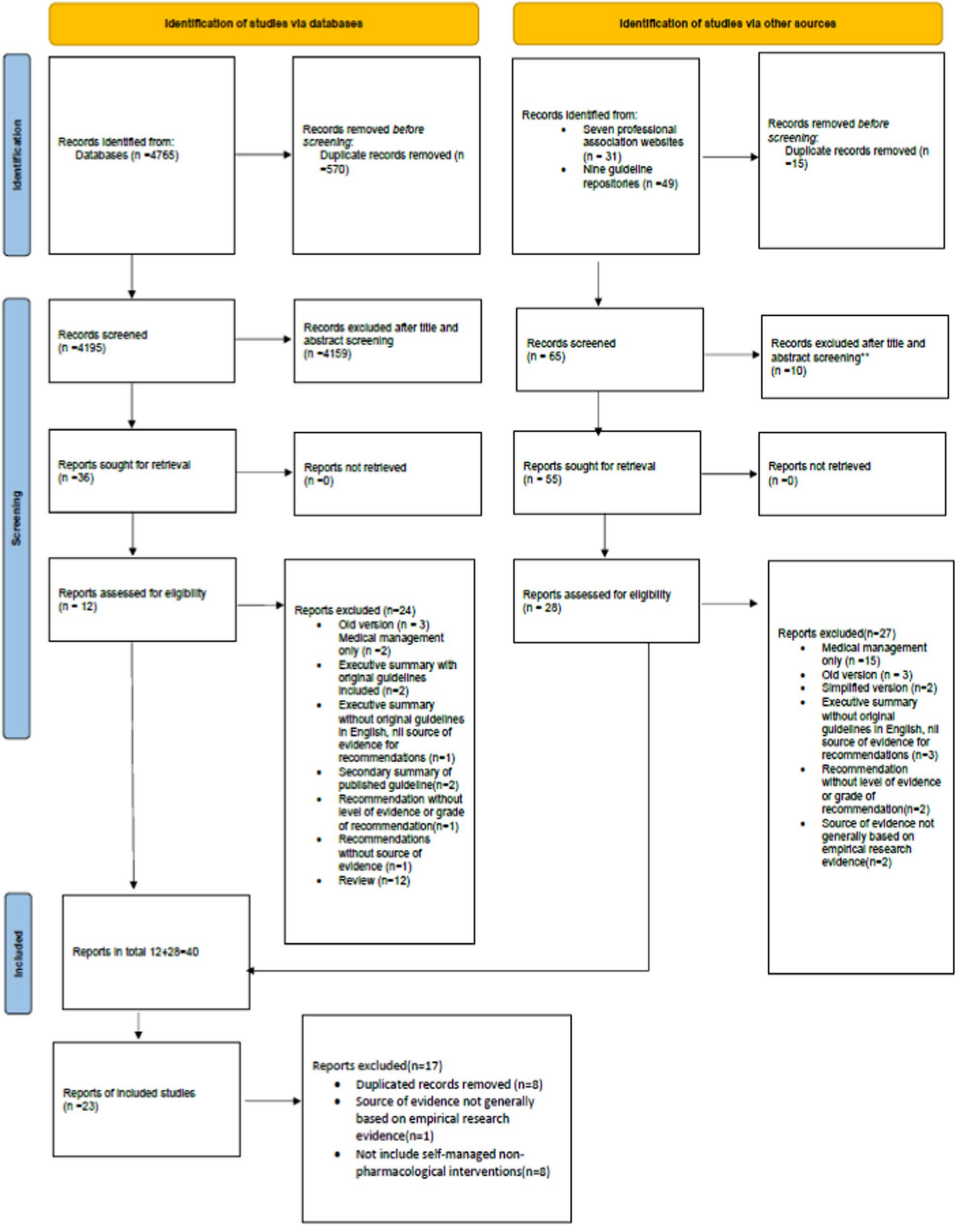
PRISMA flow diagram of study selection. Adapted from: Page MJ, McKenzie JE, Bossuyt PM, Hoffmann TC, Mulrow CD, et al. The PRISMA 2020 statement: an updated guideline for reporting systematic reviews. BMJ

The PRISMA flow chart was used as a guide and also documented with details of the selection process [37] (Fig. 1). Endnote was used to identify and remove duplicate records. Two reviewers screened the CPGs independently, a third researcher was involved in managing the disagreement and reaching consensus. Key information from the included CPGs was extracted with predefined tables: (1) the description of guideline characteristics, including title, development institution, country, year of publication/update, evidence analysis and quality tool referral (Table 2). (2) the recommendations of self-managed non-pharmacological interventions, grade of recommendation (GOR), class of recommendation (COR) and level of evidence (LOE) of each recommendation.

**Table 2:** Characteristics of the included clinical practice guidelines

Title of CPGs	Abbreviated Name	Development institution	Continent/Country	Year Published/Updated	Newly developed	Publication in a journal		Evidence analysis	Quality tool referral
						Journal name	Impact factor (2022)		
2020 AHA/ACC Guideline for the Diagnosis and Treatment of Patients with Hypertrophic Cardiomyopathy	AHA/ACC HCM (2020)	AHA/ACC	USA	2020	No	Journal of the American College of Cardiology	24.4	Systematic review, consensus method among experts, including a lay/patient representative	AHA/ACC Policies for Development of Guidelines, Performance Measures, and Data Standards, Methodology Manual and Policies From the ACCF/AHA Task Force on Practice Guidelines
2016 AHA/ACC Guideline on the Management of Patients with Lower Extremity Peripheral Artery Disease	AHA/ACC PAD (2016)	AHA/ACC	USA	2017	No	Circulation, Co-published in the Journal of the American College of Cardiology and reprinted in Vascular Medicine	- Circulation: 37.8 - Journal of the American College of Cardiology:24.4 - Vascular Medicine:3.7	Systematic literature review, a nurse in the role of patient representative in the writing committee	Methodology Manual and Policies From the ACCF/AHA Task Force on Practice Guidelines
2022 AHA ACC HFSA Guideline for the Management of Heart Failure	AHA/ACC/HFSA HF (2022)	AHA ACC HFSA	USA	2022	No	Circulation	37.8	Systematic literature review, 2 lay/patient representatives in the writing committee	Methodology Manual and Policies From the ACCF/AHA Task Force on Practice Guidelines
2019 AHA/ACC/HRS Focused Update of the 2014 AHA/ACC/HRS Guideline for the Management of Patients with Atrial Fibrillation	AHA/ACC/HRS AF (2019)	AHA ACC HRS	USA	2019	No	Journal of the American College of Cardiology, co-published in Circulation and Heart Rhythm	- Journal of the American College of Cardiology:24.4 - Circulation: 37.8	Systematic review	Methodology Manual and Policies From the ACCF/AHA Task Force on Practice Guidelines
2021 AHA ASA Guideline for the Prevention of Stroke in Patients with Stroke and Transient Ischemic Attack	AHA/ASA Stroke (2021)	AHA ASA	USA	2021	No	Stroke	8.3	Systematic Review, consensus method among experts, a lay/patient representative in the writing group	AHA/ASA Policies and Procedures for Development of Scientific Publications
2018 Australian Guidelines for the Prevention, Detection, and Management of Heart Failure	Australia HF (2018)	NHFA CSANZ	Australia	2018	No	Heart, Lung and Circulation	2.6	Systematic literature review	Not reported
2019 Brazilian CVDs prevention guideline 2019 update	Brazilian CVD (2019)	SBC	Brazil	2019	No	Arq Bras Cardiol	2.6	Systematic literature review	Not reported
2017 Comprehensive Update of the Canadian Cardiovascular Society Guidelines for the Management of Heart Failure	Canadian HF (2017)	CCS	Canada	2017	No	Canadian Journal of Cardiology	7.2	Systematic literature review	AGREE II
2020 Canadian Stroke Best Practice Recommendations Secondary Prevention of Stroke Update 2020	CSBPR (2020)	HSF Canada	Canada	2020	No	No	NA	Systematic review, consensus method among experts	AGREE II
2020 ESC Guidelines for the diagnosis and management of atrial fibrillation developed in collaboration with the European Association for Cardio-Thoracic Surgery (EACTS)	ESC AF (2020)	ESC EACTS	Europe	2020	No	European Heart Journal	39.3	Systematic review, consensus method among experts	ESC Recommendations for Guidelines Production
2021 ESC Guidelines on cardiovascular disease prevention in clinical practice	ESC CVD (2021)	ESC	Europe	2021	No	European Heart Journal	39.3	Systematic literature review	ESC Recommendations for Guidelines Production
2021 ESC Guidelines for the diagnosis and treatment of acute and chronic heart failure	ESC HF (2021)	ESC	Europe	2021	No	European Heart Journal	39.3	Systematic literature review	ESC Recommendations for Guidelines Production
2017 ESC Guidelines on the Diagnosis and Treatment of Peripheral Arterial Diseases in collaboration with the European Society for Vascular Surgery (ESVS)	ESC PAD (2017)	ESC ESVS	Europe	2018	No	European Heart Journal	39.3	Systematic literature review	ESC Recommendations for Guidelines Production
ESVM (European Journal of Vascular Medicine) Guideline on peripheral arterial disease 2019	ESVM PAD (2019)	ESVM	Europe	2019	No	Vasa-European Journal of Vascular Medicine	1.8	Systematic literature review, consensus method among experts	Not reported
JCS 2017 JHFS 2017 guideline on diagnosis and treatment of acute and chronic heart failure	JCS /JHFS HF (2017)	JCS JHFS	Japan	2017	No	Circulation Journal	3.3	Systematic literature Review	Not reported
2019 Korean Clinical Practice Guideline for Cardiac Rehabilitation in Korea Recommendations for Cardiac Rehabilitation and Secondary Prevention after Acute Coronary Syndrome	Korean CR ACS (2019)	The Korean Society of Cardiology	Korea	2019	Yes	Korean Circulation Journal	2.9	Systematic review	Not reported
2018 Malaysian clinical practice guidelines of stable coronary artery disease	Malaysia SCAD (2018)	NHAM AMM	Malaysia	2018	No	No	NA	Systematic literature review, consensus method among experts	Not reported
2019 Malaysian clinical practice guidelines management of STEMI	Malaysia STEMI (2019)	NHAM AMM	Malaysia	2019	No	No	NA	Systematic literature review, consensus method among experts	Not reported
2017 Malaysian Primary & Secondary Prevention of Cardiovascular Disease	Malaysian CVD (2017)	NHAM AMM	Malaysia	2017	Yes	No	NA	Systematic literature review, consensus method among experts	Not reported
2019 Malaysian clinical practice guidelines management of Heart Failure	Malaysian HF (2019)	NHAM AMM MOH Malaysia	Malaysia	2019	No	No	NA	Systematic literature review, consensus method among experts	Not reported
Physical Therapist Clinical Practice Guideline for the Management of Individuals with Heart Failure 2020	PT HF (2020)	APTA	USA	2020	Yes	Physical therapy	3.8	Systematic literature review, the consensus among experts, including a patient representative as external reviewer	AGREE II
2017 SIGN Risk estimation and the prevention of cardiovascular disease	SIGN 149 CVD (2017)	SIGN	UK	2017	No	No	NA	Systematic literature review	SIGN 50: a guideline developer’s handbook, 2015 edition
2019 USA VADoD clinical practice guideline for the management of stroke rehabilitation	VA/DoD Stroke Rehab (2019)	VA/DoD	USA	2019	No	No	NA	Systematic review, consensus method among experts, including patient focus group	The Guideline for Guidelines, an internal document of the VA and DoD EBPWG2019

*Note: AHA/ACC* American Heart Association/American College of Cardiology, *HFSA* the Heart Failure Society of America, *HRS* Heart Rhythm Society, *ASA* American Stroke Association, *ESC* the European Society of Cardiology, *EACTS* the European Association for Cardio-Thoracic Surgery, *ESVS* the European Society for Vascular Surgery, *ESVM* European Society for Vascular Medicine, *JCS* the Japanese Circulation Society, *JHFS* the Japanese Heart Failure Society, *NHAM* National Heart Association of Malaysia, *AMM* the Academy of Medicine Malaysia, *MOH* Malaysia, Ministry of Health Malaysia, *APTA* The American Physical Therapy Association, *SIGN* Scottish Intercollegiate Guidelines Network, *VA/DoD* Department of Veterans Affairs, Department of Defense, *CSANZ* Cardiac Society of Australia and New Zealand, *NHFA* National Heart Foundation of Australia, *HSF Canada* Heart and Stroke Foundation of Canada, *CCS* Canadian Cardiovascular Society, *SBC* Brazilian Society of Cardiology, *NA* Not applicable, *AGREE II *Appraisal of Guidelines for Research & Evaluation II

### Quality Assessment

The Appraisal of Guidelines for Research & Evaluation II (AGREE II) [38] was used by four independent experienced researchers to evaluate the quality of the included CPGs. Before the commencement of the appraisal, the four researchers read the appraisal guidelines and completed the training tools online to make sure the effective application of the AGREE II. There are 23 items in this tool, which are grouped in six domains: scope and purpose, stakeholder involvement, rigor of development, clarity and presentation, applicability, and editorial independence [38]. A 7-point Likert scale (from 1 strongly disagree to 7 strongly agree) was used to rate each item and the rigor scores of each domain were calculated according to AGREE II by four researchers independently [38]. The quality of the guideline was determined by the calculated mean percentage of the six domains. If the mean percentage was higher than 70%, the guideline was considered as high quality; if the standardised percentages were lower than 40% in more than three domains, the guideline was deemed as low quality and not recommended; if the standardised percentages were between 40 to 70% in more than three domains, the guideline was considered as moderate quality and recommended with modifications [38] (Table 3).

**Table 3:** Scores of the domains and overall assessment of the guidelines according to the AGREE II instrument

Title of guideline	Domain scores (%)							Overall quality	Degree of recommendation	ICC
	Scope and	Stakeholder involvement	Rigor of development	Clarity and presentation	Applicability	Editorial	Average			
	Purpose					Independence				
JCS /JHFS HF (2017)	76.4	75	53.1	61.1	51	72.9	64.9	Moderate	RM	0.775
Brazilian CVD (2019)	70.8	61.1	54.7	75	58.3	70.8	65.1	Moderate	RM	0.822
Canadian HF (2017)	83.3	72.2	54.2	91.7	66.7	47.9	69.3	Moderate	RM	0.827
ESC CVD (2021)	61.1	77.8	56.3	86.1	56.3	100	72.9	High	R	0.797
ESC PAD (2017)	72.2	76.4	57.3	86.1	59.4	100	75.2	High	R	0.853
ESC HF (2021)	75	68.1	56.3	90.3	65.6	97.9	75.5	High	R	0.843
ESC AF (2020)	76.4	72.2	55.7	88.9	61.5	100	75.8	High	R	0.853
CSBPR (2020)	87.7	70.8	58.9	70.8	80.2	95.8	77.4	High	R	0.812
VA/DoD Stroke Rehab (2019)	88.9	77.8	90.1	84.7	64.5	77.1	80.5	High	R	0.806
ESVM PAD (2019)	88.9	75	65.6	100	57.3	100	81.1	High	R	0.894
Korean CR ACS (2019)	90.3	90.3	81.8	80.6	66.7	100	84.9	High	R	0.765
Australia HF (2018)	90.3	95.8	76.6	76.8	84.4	100	87.3	High	R	0.8
AHA/ACC HCM (2020)	87.5	90.3	99.5	100	67.7	100	90.8	High	R	0.815
PT HF (2020)	97.2	93.1	95.3	98.6	71.9	97.9	92.3	High	R	0.75
ACC/AHA PAD (2016)	98.6	90.3	99.5	100	69.8	95.8	92.3	High	R	0.812
Malaysian CVD (2017)	98.6	87.5	84.9	98.6	100	97.9	94.6	High	R	0.777
Malaysia STEMI (2019)	98.6	87.5	86.9	98.6	100	100	95.3	High	R	0.703
AHA/ACC/HRS AF (2019)	100	88.9	99.5	97.2	88.5	97.9	95.3	High	R	0.732
AHA/ASA Stroke (2021)	98.6	100	99.5	100	75	100	95.5	High	R	0.855
Malaysian HF (2019)	100	87.5	90.6	100	100	97.9	96	High	R	0.795
SIGN 149 CVD (2017)	95.8	97.2	95.8	98.6	89.6	100	96.2	High	R	0.556
Malaysia SCAD (2018)	100	91.7	90.6	100	100	100	97.1	High	R	0.651
AHA/ACC/HFSA HF (2022)	95.8	91.7	98.9	97.2	100	100	97.3	High	R	0.765
Average	88.35	83.4	78.33	90.47	75.41	93.47	84.9	High	R	0.785

*ICC* interclass correlation coefficient, *R* Recommended, *RM* Recommended with modifications

### Data synthesis and analysis

Intraclass correlation coefficient (ICC) was used to examine internal consistency among the four assessors in the quality assessment of the included CPGs [39]. The ICC value ranges from 0–1, with the value less than 0.5 indicating poor reliability, 0.5 to 0.75 indicating moderate reliability, 0.75 to 0.9 and more than 0.9 indicating good and excellent reliability, respectively [39]. ICC was calculated and analysed by SPSS 26.0, the 95% confidence interval of the ICC estimate was used to determine the degree of reliability [39]. The domain scores, the mean percentage of the six domains, the overall quality, the degree of recommendations and ICC of each included CPG were listed in Table 2.

Descriptive analysis was adopted to summarise and categorize the self-managed non-pharmacological interventions recommended from the included  CPGs. The recommendations were extracted and summarized in different tables according to different CVDs (Table 4–11). For each CVD, the recommendations were categorized according to different self-managed non-pharmacological interventions, including healthy diet, physical activity, weight management, smoking cessation and others. Class of Recommendation (COR) and level of evidence (LOE) of each recommendation were listed and analysed as well.

## Results

There were 4765 results generated from databases and 80 results from professional association websites (n = 31) and guideline repositories (n = 49). Results from databases and other sources were checked and screened separately. 40 reports (databases 12, other sources 28) were left after initial screening, and 17 reports were further excluded with reasons. As a result, there were a total of 23 clinical practice guidelines (CPGs) developed or updated between 2017 and April 2022 were included (Fig. 1).

### Characteristics of the included CPGs

Guidelines regarding different cardiovascular diseases were included, in which four guidelines of cardiovascular diseases [17–20], three for coronary heart diseases [21, 40, 41], seven for heart failure [22, 42–47], two for atrial fibrillation [48, 49], three for stroke [50–52], three for peripheral arterial disease [31, 53, 54], and one for hypertrophic cardiomyopathy [55]. Seven of these CPGs originated from the United States of America [22, 31, 43, 50, 52, 55], five from Europe [17, 42, 48, 53, 54], four from Malaysia [19, 40, 41, 44], two from Canada [47, 51], one each from the United Kingdom [18], Korea [21], Japan [45], Australia [46], and Brazil [20]. Three of the 23 CPGs were newly developed [19, 21, 43], and the remaining twenty-one guidelines were newly updated. Sixteen of the 23 were published in a journal, while five [19, 40, 41, 44, 52] were published in The Turning Research Into Practice (TRIP) database, one [18] was published in The Scottish Intercollegiate Guidelines Network, one [51] was published in Heart and Stroke Foundation of Canada as official documents.

All twenty-three CPGs used a systematic review approach to provide recommendations with the highest level of evidence based on the analysis of experts, six of these CPGs involved patients in the guideline development [22, 31, 43, 50, 52, 55]. Regarding quality tool referral, ten CPGs (43.5%) did not report specific quality tools, the other thirteen CPGs used different quality tools. Two Canadian CPGs used AGREE II instrument [47, 51], four ESC CPGs referred to ESC Recommendations for Guidelines Production [17, 42, 48, 54], SIGN 149 referred to SIGN 50: a guideline developer’s handbook (2015 edition), VA/DoD Stroke Rehab (2019) referred to the Guideline for Guidelines which is an internal document for the VA and DoD EBPWG which was updated in January 2019 [52], one AHA/ASA Stroke guideline used AHA/ASA policies and methods for the development of guidelines [50], four AHA/ACC CPGs referred to Methodology Manual and Policies from the ACCF/AHA Task Force on Practice Guidelines [22, 31, 49, 55] (Table 2).

### Quality appraisal of included CPGs

The average ICC for each guideline between four reviewers was 0.785, ranged from 0.556 to 0.894, in which 19 guidelines ranged between 0.75 to 0.90 indicating good reliability (Table 3). Overall, the ICC suggested moderate to high consistency of rating scores among four reviewers. The AGREE II domain scores for each guideline varied from 47.9% to 100%. The domain of “Editorial Independence” had the highest mean domain score of 93.47% (range: 47.9 -100%), whereas the domain of “Applicability” had the lowest mean domain score of 75.41% (range: 51–100%), see Table 3. The domain of “Editorial independence” refers to the conflicts of interest and funding sources, which had the highest mean domain score of 93.47% (range: 47.9 -100%). This result suggested high credibility and reliability of the included CPGs given the transparent and unbiased development process. The domain of “Rigor of Development” was the second lowest among the evaluated domains with the mean domain score 78.33% (range: 53.1–99.5%), which suggested the robustness and trustworthiness of the included CPGs were relatively low. Notably, there were eight CPGs with this domain score of less than 60%, it highlighted that careful consideration will be needed when utilising the recommendations from these CPGs [17, 20, 42, 45, 47, 48, 51, 54]. The domain of “Applicability” had the lowest mean domain score of 75.41% (range: 51–100%), which indicated potential variability in the included CPG’s articulation of facilitators and barriers to the application of the recommendations, also the adaptability of the real-world implementation of the recommendations. There were five CPGs with the domain score of less than 60%, careful consideration for utilisation will be needed as well from these CPGs [17, 20, 45, 53, 54].

According to AGREE II instrument, the average percentage of the six domains ranged from 64.9% to 97.3%, with the mean overall standardised percentage 84.9%. According to predefined standard, twenty CPGs with a mean percentage over 70% were rated as “recommended” (high quality). The other three CPGs [20, 45, 47] were rated as “recommended with modification” (moderate quality, standardized percentages were between 40 and 70% in over three domains). Therefore, all these 23 CPGs were recommended for use with or without modification.

### Summary of self-managed non-pharmacological interventions

The included 23 CPGs recommended self-managed non-pharmacological interventions for patients with CVDs. Details of these recommendations for different diseases are summarised in Table 4–11.

**Table 4:** Diet recommended by the included CPGs for CVDs and CHD

Nutrition composition and food groups	Detailed nutritional profile & food categories	ESC CVD 2021	Malaysian CVD 2017	SIGN 149 2017	Brazilian CVD 2019	Korean 2019 CR
Nutrition composition	Fat		Aim for 20–25% (max 30%) of energy from fat, with 7–10% from saturated fatty acids (SFA). Substitute SFA with monounsaturated (MUFA) and polyunsaturated fatty acids. COR/LOE: I/B	Recommend low in saturated fats diet. COR/LOE: R/1 + + Daily saturated fat: men ≤ 30 g, women ≤ 20 g COR/LOE: ✔/4		Limit total fat lower than 30% of energy intake, saturated fat to 7%, and omega-6 PUFA to 10%. Restrict trans-fat less than 1%. COR/LOE: GPP/2 +
Nutrition composition	Trans fatty acid	Minimize trans unsaturated fatty acids, especially from processed foods. Reduce red meat to 350-500 g weekly, and limit processed meat. COR/LOE: I/A	Keep trans fatty acid (TFA) below 1% and minimize high-fat processed meat and bakery products. Reduce partially hydrogenated fats. COR/LOE: I/A			
Nutrition composition	Cholesterol		No strict restrictions on cholesterol-rich foods/eggs. Limit cholesterol intake to < 200 mg/day for high CV risk individuals. Be cautious about SFA content in cholesterol-rich foods. COR/LOE: IIa/B			Limit cholesterol intake < 300 mg/day. COR/LOE: GPP/2 +
Nutrition composition	Protein		10–20% of total energy intake. COR/LOE: I/B			
Nutrition composition	Carbohydrate (CHO)		50–60% of total energy intake. Recommend High-fiber, complex carbohydrates from whole grains, fruits, and vegetables COR/LOE: I/B Limit sugar intake to 5–10% of energy intake. COR/LOE: I/A			
Food groups	Dietary fiber	Opt for a fiber-rich diet with whole grains, fruits, vegetables, pulses, and nuts. Aim for 30-45 g of fiber per day. COR/LOE: I/A	Aim for 20-30 g daily from various sources. COR/LOE: I/B			Ensure daily fiber intake is above 25 g. COR/LOE: GPP/2 +
Food groups	Whole grain	Fiber 30–45 g/day of, preferred wholegrains. COR/LOE: I/A	Recommend half grain intake from the whole grain. COR/LOE: I/B			
Food groups	Fruits and vegetables	Consume at least 200 g of fruits daily, which is equivalent to 2–3 servings, and also aim for a minimum of 200 g of vegetables daily, again equivalent to 2–3 servings. COR/LOE: I/A	fruits and vegetables five servings/day. COR/LOE: I/B	Increase fruit and vegetable consumption COR/LOE: R/2 + + , 2 +		
Food groups	Nuts	30 g unsalted nuts per day. COR/LOE: I/A	30-g unsalted nuts per day. COR/LOE: IIa/B	Insufficient evidence to support a recommendation.		
Food groups	Salt	< 5 g total salt intake per day. COR/LOE: I/A	daily salt consumption less than 5 g or 1 level teaspoon or (2000 mg sodium). COR/LOE: I/A	Advise those with hypertension to minimize salt intake to lower blood pressure. COR/LOE: R/1 + Limit daily salt intake less than 6 g. COR/LOE: ✔/4		Limit daily intake of salt less than 5 g (or sodium less than 2 g) COR/LOE: GPP/2 +
Food groups	Sugar	Limit free sugar intake, especially sugar-sweetened beverages, to max 10% of energy intake. COR/LOE: I/B	Less than 10% of total energy from added sugar. COR/LOE: I/A			Restrict added sugar to 10% of total energy intake. COR/LOE: GPP/2 +
Food groups	Alcohol	Limit maximum alcohol consumption to 100 g/week. COR/LOE: I/B	For non-pregnant women, limit alcohol to 1 drink (10 g/day) and men to 2 drinks a day. COR/LOE: IIa/ B	Reduce alcohol consumption. COR/LOE: R/2 + + , 2 + , 2-		Ideally, avoid alcohol; if not, limit to less than 20mgfor men and 10 mg for women. (1shot 10 mg) COR/LOE: GPP/2 +
Specific minor dietary components	Fish and Omega-3 Supplementation	Fish 1–2 times per week, particularly fatty fish. COR/LOE: I/A	Fresh fish is preferred, and deep-frying should be avoided. COR/LOE: IIa/ B	Have fish more than two portions/week, oily fish for at least once(one portion 140 g). COR/LOE: ✔/4	Eat two fish meals per week, particularly for individuals at high risk. COR/LOE: I/B	
Specific minor dietary components	Plant based foods rich in omega-3 fatty acids				Encourage the consumption of omega-3 polyunsaturated fatty acids of plant origin. COR/LOE: IIb/B	
Dietary pattern	Dietary pattern	Mediterranean or similar diet. COR/LOE: I/A Prioritize plant-based, fiber-rich foods such as fruits, vegetables, pulses, whole grains, and nuts. COR/LOE: I/B	Malaysian Healthy Plate and Current Healthy Eating Recommendation •More plant-based foods such as nuts, legumes, beans, fruits and vegetables • More whole grain foods • More fish •low-fat dairy products • Healthy oils • Food with less reduced sweetness • Less processed /salty foods. COR/LOE: I/B	A Mediterranean dietary pattern enriched with an additional 30 g/day of either extra virgin olive oil or unsalted nuts. COR/LOE: R/1 + + , 1 + , 2 + + Utilize the Eatwell Guide to assist people in making well-informed decisions about their dietary choices, focusing on selecting appropriate food components in the right proportions. This approach aligns with the Mediterranean diet model, while also limiting the intake of saturated fat, sugars, and salt. COR/LOE: ✔/4		Ensure a balanced intake of various food groups while consuming the right amount of energy to support a healthy weight. This includes incorporating whole grains, vegetables, fruits, fish poultry, beans, and nuts into your diet COR/LOE: GPP/2 +

COR, Class of Recommendation; LOE, Level of Evidence

### Cardiovascular disease

Four CPGs on prevention of cardiovascular disease were included [17–20]. These CPGs provided comprehensive recommendations including diet, physical activity, weight management and smoking cessation (Table 4 and 5).

**Table 5:** Non-pharmacological interventions recommended by the included CPGs for CVDs

Intervention types	Recommended self-managed non-pharmacological interventions	COR/LOE	Grading system used	Guidelines
Physical Activity	Aim for moderate intensity aerobic physical activity 150–300 min/week, or vigorous intensity aerobic activity 75-150 min/week, or a mix of both that provides an equivalent level of exercise Form: moderate or vigorous intensity aerobic PA Duration/Frequency: 150–300 min or 75–150 min/week	I/A	ESC Guidelines Classification Scheme	ESC CVD (2021)
Physical Activity	For adults who are unable to engage in the moderate-intensity physical activity for 150 min/week, it is recommended to remain as active as their physical capabilities and health status permit Form: NR Duration/Frequency: NR	I/B	ESC Guidelines Classification Scheme	ESC CVD (2021)
Physical Activity	To minimize sedentary time, engaging in at least light activity during the day. Form: light activity Duration/Frequency: NR	I/B	ESC Guidelines Classification Scheme	ESC CVD (2021)
Physical Activity	Recommend resistance exercise, in addition to aerobic activity for at least 2 days/week for reducing all-cause mortality. Form: resistance exercise, in addition to aerobic activity Duration/Frequency: more than 2 days per week	I/B	ESC Guidelines Classification Scheme	ESC CVD (2021)
Physical Activity	Recommend moderate-intensity PA for the whole population unless contraindicated. Enhancing activity levels can be achieved by adjusting the intensity, duration, or frequency of the activity. Individuals should minimize sedentary time, particularly over extended periods. Form: Moderate-intensity PA, minimize sedentary time Duration/Frequency: NR	R/2 + + , 2 +	SIGN criteria	SIGN 149 CVD (2017)
Physical Activity	Recommend PA including occupational and/or leisure-time activities, such as brisk walking. Form: occupational and/or leisure-time activities Duration/Frequency: NR	R/2 + + , 2 +	SIGN criteria	SIGN 149 CVD (2017)
Physical Activity	Encourage individuals who are moderately active and capable of boosting their physical activity to do so by making adjustments to the intensity, duration, or frequency of their activities Form: NR Duration/Frequency: NR	R/2 + + , 2 +	SIGN criteria	SIGN 149 CVD (2017)
Physical Activity	Individuals should minimize sedentary time, particularly over extended periods. Form: NR Duration/Frequency: NR	R/2 + + , 2 +	SIGN criteria	SIGN 149 CVD (2017)
Physical Activity	Encourage all patients to increase activity levels gradually, regardless of their current health, fitness, or activity level. Form: NR Duration/Frequency: NR	✔/4	SIGN criteria	SIGN 149 CVD (2017)
Physical Activity	Recommend exercise with moderate intensity for at least 150 min/week or more intense exercise for 75 min/week to reduce cardiovascular risk. Form: moderate intensity exercise or more intense exercise Duration/Frequency: at least 150 min/week or 75 min/week	I/A	Brazilian Society of Cardiology (SBC) criteria	Brazilian CVD (2019)
Physical Activity	Doing moderate intensity exercise < 150 min/week or more intense exercise < 75 min/week still reduces cardiovascular risk. Form: moderate intensity exercise or more intense exercise Duration/Frequency: less than 150 min/week or 75 min/week	IIa/B	SBC criteria	Brazilian CVD (2019)
Physical Activity	The suggested amount of PA for healthy adults, regardless of age, is moderate-intensity exercise 150 min/week, or vigorous-intensity exercise 75 min/week. Alternatively, they can opt for a combination of both. Additionally, it is advisable to incorporate resistance exercises on more than two days/ week and flexibility exercises on 2–3 days/ week, whenever feasible or required. Form: moderate intensity, vigorous intensity, resistance exercise, flexibility exercise Duration/Frequency: at least 150 min/week, 75 min/week, 2 days/week, 2–3 days/week	I/B	ACC/AHA Clinical Practice Guideline Recommendation Classification System and ESC Guidelines Classification Scheme	Malaysian CVD (2017)
Smoking cessation	All tobacco use should be stopped, as it is a strong and independent cause of ASCVD.	I/A	ESC Guidelines Classification Scheme	ESC CVD (2021)
Smoking cessation	Regardless of weight gain, it is advisable to quit smoking since the advantages of cessation for ASCVD remain unaffected by weight fluctuations.	I/B	ESC Guidelines Classification Scheme	ESC CVD (2021)
Smoking cessation	Advise all smokers to stop and offersupport to minimize cardiovascular and general health risks.	R/2 + + , 2 + , 4	SIGN criteria	SIGN 149 CVD (2017)
Smoking cessation	Exposure to passive smoking should be minimized, as it increases cardiovascular risk.	R/2 + + , 2 +	SIGN criteria	SIGN 149 CVD (2017)
Smoking cessation	Recommend smoking cessation for all adults to decrease cardiovascular risk.	I/B	SBC criteria	Brazilian CVD (2019)
Smoking cessation	Complete cessation: a combination of physiological and psychological intervention. Avoid exposure to second-hand tobacco smoke.	I/B	ACC/AHA Clinical Practice Guideline Recommendation Classification System and ESC Guidelines Classification Scheme	Malaysian CVD (2017)
Smoking cessation	E-cigarettes and shisha use are not recommended.	III/B	ACC/AHA Clinical Practice Guideline Recommendation Classification System and ESC Guidelines Classification Scheme	Malaysian CVD (2017)
Weight management	Overweight and obese individuals should aim to reduce weight to improve their cardiovascular health. Form: Healthy diet	I/A	ESC Guidelines Classification Scheme	ESC CVD (2021)
Weight management	Weight reduction interventions should aim for at least a 3 kg weight loss and maintenance.	R/2 + + , 2 + , 1 + + , 1 +	SIGN criteria	SIGN 149 CVD (2017)
Weight management	Weight should be measured annually.	✔/ gpp	SIGN criteria	SIGN 149 CVD (2017)
Weight management	Overweight and obese individuals should aim for weight loss to improve their cardiovascular risk profile.	I/B	SBC criteria	Brazilian CVD (2019)
Weight management	Overweight and obese individuals should receive counseling and interventions aimed at achieving and maintaining weight loss, including caloric restriction and lifestyle modifications.	I/B	SBC criteria	Brazilian CVD (2019)
Weight management	The goals of weight management therapy are to achieve 5–10% weight loss and maintain it for 1–2 years before attempting further weight loss.	I/B	ACC/AHA Clinical Practice Guideline Recommendation Classification System and ESC Guidelines Classification Scheme	Malaysian CVD (2017)
Weight management	Recommended waist circumference thresholds for evaluating abdominal obesity are as follows: Less than 90 cm for men and less than 80 cm for women.	I/A	ACC/AHA Clinical Practice Guideline Recommendation Classification System and ESC Guidelines Classification Scheme	Malaysian CVD (2017)
Weight management	Recommended Dietary Weight-Loss Strategies: Practical initial target for weight loss is a negative deficit of 500 cal per day, with greater weight loss requiring a calorie restriction of 1200 to 1500 kcal per day achieved through meal replacement or calorie counting.	I/B	ACC/AHA Clinical Practice Guideline Recommendation Classification System and ESC Guidelines Classification Scheme	Malaysian CVD (2017)
Weight management	Recommended PA Weight-Loss Strategies: For unfit individuals, PA should be started slowly and gradually increased each week, such as starting at 60 min per week and slowly increasing to 150 min per week. To achieve weight loss, it is recommended to do moderate-intensity PA 250-450 min/week, which should also include strength training sessions of 2 to 3 times/ week.	I/B	ACC/AHA Clinical Practice Guideline Recommendation Classification System and ESC Guidelines Classification Scheme	Malaysian CVD (2017)
Weight management	Recommended Behavioral Weight-Loss Strategies: Various behavioral strategies, such as self-monitoring of dietary patterns and PA, are necessary to maintain weight loss, but this method may yield only small reductions in body weiht in primary care settings.	I/B	ACC/AHA Clinical Practice Guideline Recommendation Classification System and ESC Guidelines Classification Scheme	Malaysian CVD (2017)

Note: PA, physical activity; NR, not reported; COR, Class of Recommendation; LOE, Level of Evidence

#### Diet

Three CPGs provided comprehensive diet recommendations including nutritional composition of food, dietary patterns, and food groups for the prevention and management of CVD with high to moderate LOE [17–19], while one CPG only recommended specific minor dietary components with LOE and GOR [20] (Table 4). Regarding nutritional composition, three CPGs recommended low saturated fat [17–19], in which one CPG did give detailed suggestions about the intake of unsaturated fatty acid, trans fatty acid, cholesterol, carbohydrates, and protein [19].

Regarding dietary pattern, three CPGs recommended Mediterranean or similar diet encouraging more plant, less animal-based food [17–19]. Regarding food groups, three CPGs encouraged fruits and vegetables intake, in which two CPGs recommended 30 g unsalted nuts, limit salt less than 5 g/day and sugar to less than 10% of total energy intake [17, 19], while one CPG recommended salt intake less than 6 g/day for all individuals and reduce salt intake as much as possible for patients with hypertension and no suggestions for nuts or sugar [18]. Three CPGs gave suggestions regarding alcohol consumption, for example, a maximum of 100 g/week [17], abstinence or less than 1–2 standard drink/day [19]. Fish was recommended by all four CPGs, two CPGs stated that Omega-3 should not be used in reducing CVD risk [18, 19], while one CPG suggested to use Omega-3 supplementation for the secondary prevention [20].

#### Physical activity

All four CPGs highly recommended physical activity with moderate to high quality evidence [17–20]. One CPG suggested at least moderate physical activity without information related to the frequency or duration [18], while the other three CPGs made the same strong recommendations of moderate intensity activity at least 150 min/week or vigorous intensity activity 75 min/week with high quality evidence. One CPG did give another suggestion with less than 150 min moderate intensity activity or 75 min more intense exercise with moderate LOE(B) [20]. Similarly, another CPG advised patients to stay as active as allowed by health condition and ability if they cannot perform moderate intensity activity 150 min/week with moderate LOE(B) [17]. In addition to aerobic training, resistance exercise for more than 2 days/week were recommended by two CPGs [17, 18], while one CPG also suggested flexibility exercise 2–3 days/week [19]. Sedentary time was strongly encouraged to minimise by two CPGs and suggested to engage in at least light activity [17, 18].

#### Smoking cessation

All four CPGs highly recommended smoking cessation with moderate to high quality evidence, but no detailed self-managed non-pharmacological smoking cessation interventions given. Avoiding second-hand smoking was suggested by two CPGs [18, 19].

#### Weight management

Weight management for overweight and obese patients were highly recommended by four CPGs with moderate to high quality evidence. Two CPGs recommended the goal of weight loss, one CPG suggested to reduce weight by at least 3% and maintain this reduction, and suggested patients to measure weight annually [18], while another set the aim of 5–10% and maintain this reduction over 1–2 years before trying further loss and set the cut-offs of waist circumference less than 90 cm for men and less than 80 cm for women [19]. Three CPGs recommended non-pharmacological interventions to help achieve the goal of weight management, including diet (e.g., calories restriction) [19, 20], physical activity [19], and behavioural modifications (e.g., self-monitoring of eating habits) [17, 19]. Only one CPG did not specify weight loss interventions [18].

### Coronary Heart Disease (CHD)

Three CPGs were included for the management of coronary heart disease (CHD), recommendations include diet, physical activity, weight management and smoking cessation (Table 6) [21, 40, 41].

**Table 6:** Non-pharmacological interventions recommended by the included CPGs for CHD

Intervention types	Recommended self-managed non-pharmacological interventions	COR/LOE	Grading system used	Guidelines
Diet	Details listed in diet Table 4	GPP/2 +	SIGN criteria 2017	Korean CR ACS (2019)
Diet	Refer to Malaysian CVD (2017)			Malaysia STEMI (2019)
Diet	Refer to Malaysian CVD (2017)			Malaysia SCAD (2018)
Weight management	The goal is to attain a weight reduction of 5 to 10% and sustain it for 1 to 2 years prior to pursuing additional weight loss efforts. Approaches to achieve this include dietary adjustments, increased PA, behavioral changes, medication usage, and bariatric surgery.	I/B	ACC/AHA Clinical Practice Guideline Recommendation Classification System and ESC Guidelines Classification Scheme	Malaysia SCAD (2018)
Weight management	Maintaining a healthy weight or losing weight.	I/B	ACC/AHA Clinical Practice Guideline Recommendation Classification System and ESC Guidelines Classification Scheme	Malaysia STEMI (2019)
Physical activity	Exercise therapy for cardiac rehab. Form: aerobic exercise, resistance/strengthening exercises. Duration/Frequency: NR	Strong/ + +	SIGN criteria 2017	Korean CR ACS (2019)
Physical activity	Home-based cardiac rehabilitation (CR) programs could potentially serve as substitutes for hospital-based CR programs among low-risk patients. Form: NR Duration/Frequency: NR	Conditional/1 + +	SIGN criteria 2017	Korean CR ACS (2019)
Physical activity	CR programs should also be provided to patients aged 65 years and older. Form: NR Duration/Frequency: NR	Strong/1 + +	SIGN criteria 2017	Korean CR ACS (2019)
Physical activity	Form: Moderate or vigorous intensity PA Duration/Frequency: moderate intensity PA at least 150 min/week or vigorous intensity PA 75 min/week or an equivalent combination	I/B	ACC/AHA Clinical Practice Guideline Recommendation Classification System and ESC Guidelines Classification Scheme	Malaysia SCAD (2018)
Physical activity	Encourage sedentary patients to start light-intensity exercise programs after a proper exercise-related risk assessment Form: Light intensity exercise program Duration/Frequency: NR	I/B	ACC/AHA Clinical Practice Guideline Recommendation Classification System and ESC Guidelines Classification Scheme	Malaysia SCAD (2018)
Physical activity	Recommend all individuals to perform PA , and any amount of PA is beneficial for health. Form: Any Duration/Frequency: Any amount	I/B	ACC/AHA Clinical Practice Guideline Recommendation Classification System and ESC Guidelines Classification Scheme	Malaysia SCAD (2018)
Physical activity	Refer to Malaysian CVD (2017)	I/A	ACC/AHA Clinical Practice Guideline Recommendation Classification System and ESC Guidelines Classification Scheme	Malaysia STEMI (2019)
Smoking cessation	Smoking cessation	Strong/1 + +	SIGN criteria 2017	Korean CR ACS (2019)
Smoking cessation	Smoking cessation	I/A	ACC/AHA Clinical Practice Guideline Recommendation Classification System and ESC Guidelines Classification Scheme	Malaysia STEMI (2019)
Smoking cessation	Avoid second-hand tobacco smoke exposure	I/B	ACC/AHA Clinical Practice Guideline Recommendation Classification System and ESC Guidelines Classification Scheme	Malaysia SCAD (2018)
Smoking cessation	E-cigarettes and shisha should not be used as they are harmful	III/B	ACC/AHA Clinical Practice Guideline Recommendation Classification System and ESC Guidelines Classification Scheme	Malaysia SCAD (2018)

PA, physical activity; NR, not reported; COR, Class of Recommendation; LOE,Level of Evidence

#### Diet

Two CPGs of Malaysia did ask clinicians to refer to Malaysian CVD (2017) [19] for dietary recommendations and the dietary recommendations summarized in the results of CVD and listed in Table 4 [40, 41]. The other CPG recommended diet programs should be designed with reference to the diet program proposed by Korean Society of Clinical Nutrition for CR patients [21] (listed in Table 4). This diet program encouraged patients to have diversity food, eat enough grains and vegetables, limit added sugar less than 10% of total energy intake, and limit salt less than 5 g [21]. But it did not recommend food supplements with conditional strength of recommendation and LOE 4 (expert opinion) [21].

#### Weight management

Two CPGs gave some recommendations about weight management with moderate evidence [40, 41]. One guideline did not give any details of weight control [21], while the other one set the goal and offered methods of weight loss [41]. This guideline recommended patients with overweight or obesity to set the weight loss goal at 5%-10% and maintain this weight for over 1–2 years, after which they can attempt to achieve more weight loss [41]. The suggested methods of weight loss that patients can self-manage at home include increased physical activity, healthy diet, and some behavioural modifications such as self-monitoring eating habits and physical activity [41].

#### Physical activity

One guideline did not provide any self-managed physical activities but ask clinicians to refer to Malaysian CVD (2017) [19] for details (summarized in results of CVD) [40]. The other two CPGs did give some recommendations about physical activity [21, 41]. One CPG encouraged all patients to do some exercise, suggested physical activities including moderate activity for at least 150 min/week or vigorous activity for 75 min/week or an equivalent combination [41]. Another CPG recommended exercise therapy for cardiac rehabilitation, low risk patients can do home-based cardiac rehabilitation (CR) programs programs [21].

#### Smoking cessation

All three CPGs strongly recommended smoking cessation with moderate to high level of evidence. Only one guideline suggested not to use e-cigarettes or shisha, also to avoid exposure to second-hand tobacco smoke [41]. Malaysia STEMI (2019) suggested to refer to Malaysian CVD (2017) for details (listed in Table 5).

### Heart failure

There were seven CPGs of heart failure (HF) included [22, 42–47]. The recommendations include prevention and management of heart failure (Table 7).

**Table 7:** Non-pharmacological interventions recommended by the included CPGs for of HF

Prevention & management	Recommended self-managed non-pharmacological interventions	COR/LOE	Grading system used	Guidelines
Prevention of HF	Embracing a wholesome way of living, which includes consistent PA, keeping a balanced weight, practicing nutritious eating habits, and abstaining from smoking, can lower the potential likelihood of heart failure among the broader populace in the times to come.	1/B-NR	ACC/AHA Clinical Practice Guideline Recommendation Classification System (Updated May 2019)	AHA/ACC/HFSA HF (2022)
Prevention of HF	PA is recommended for all individuals to minimize the likelihood of developing HF.	Strong/Moderate	GRADE standards	Canadian HF (2017)
Prevention of HF	Smoking cessation	Strong/Low	GRADE methodology	Australia HF (2018)
Prevention of HF	Avoiding excess alcohol	Strong/ very low	GRADE methodology	Australia HF (2018)
Prevention of HF	Weight reduction for overweight or obese patients	Strong/low	GRADE methodology	Australia HF (2018)
Prevention of HF	Regular PA	Strong/low	GRADE methodology	Australia HF (2018)
Prevention of HF	Healthy lifestyles	I/B	ACC/AHA Clinical Practice Guideline Recommendation Classification System and ESC Guidelines Classification Scheme	Malaysian HF (2019)
Prevention of HF	Smoking cessation	I/B	ACC/AHA Clinical Practice Guideline Recommendation Classification System and ESC Guidelines Classification Scheme	Malaysian HF (2019)
Prevention of HF	PA Regular exercise Form: Moderate intense activity Duration/Frequency: At least 150 min/week	I/B	ACC/AHA Clinical Practice Guideline Recommendation Classification System and ESC Guidelines Classification Scheme	Malaysian HF (2019)
Prevention of HF	Maintain ideal body weight	I/B	ACC/AHA Clinical Practice Guideline Recommendation Classification System and ESC Guidelines Classification Scheme	Malaysian HF (2019)
Prevention of HF	Reducing alcohol consumption	I/C	ACC/AHA Clinical Practice Guideline Recommendation Classification System and ESC Guidelines Classification Scheme	Malaysian HF (2019)
Prevention of HF	General lifestyle modifications via weight reduction and enhanced PA.	I/A, A/I	COR and LOE similar to those used in ACC/AHA guidelines and the ECS guidelines, and MINDS	JCS /JHFS HF (2017)
Prevention of HF	Smoking cessation	I/C, B/Ivb	COR and LOE similar to those used in ACC/AHA guidelines and the ECS guidelines, and MINDS	JCS /JHFS HF (2017)
Prevention of HF	Control alcohol consumption	IIa/C, C1/VI	COR and LOE similar to those used in ACC/AHA guidelines and the ECS guidelines, and MINDS	JCS /JHFS HF (2017)
Prevention of HF	PA and exercise habits	I/B, B/Iva	COR and LOE similar to those used in ACC/AHA guidelines and the ECS guidelines, and MINDS	JCS /JHFS HF (2017)
Management of HF				
Diet and nutrition, fluid restriction	Well- balanced diet, avoid adding salt or flavoring sauces.	IIa/B	ACC/AHA Clinical Practice Guideline Recommendation Classification System and ESC Guidelines Classification Scheme	Malaysian HF (2019)
Diet and nutrition, fluid restriction	Individualized fluid intake, 1–1.5 L/day for patients with normal renal function.	IIa/C	ACC/AHA Clinical Practice Guideline Recommendation Classification System and ESC Guidelines Classification Scheme	Malaysian HF (2019)
Diet and nutrition, fluid restriction	Avoiding excessive sodium intake for stage C HF patients.	2a/C-LD	ACC/AHA Clinical Practice Guideline Recommendation Classification System (Updated May 2019)	AHA/ACC/HFSA HF (2022)
Diet and nutrition, fluid restriction	Fluid restriction: uncertain benefit for patients with advanced HF and hyponatremia.	2b/C-LD	ACC/AHA Clinical Practice Guideline Recommendation Classification System (Updated May 2019)	AHA/ACC/HFSA HF (2022)
Diet and nutrition, fluid restriction	Low-salt diet (< 6 g/day)	IIa/C, C1/VI	COR and LOE similar to those used in ACC/AHA guidelines and the ECS guidelines, and MINDS	JCS /JHFS HF (2017)
Diet and nutrition, fluid restriction	Restriction of alcohol (Moderate in drinking)	IIa/C, C1/VI	COR and LOE similar to those used in ACC/AHA guidelines and the ECS guidelines, and MINDS	JCS /JHFS HF (2017)
Diet and nutrition, fluid restriction	Restrict dietary salt intake to 2-3 g/day	Weak/Low	GRADE standards	Canadian HF (2017)
Diet and nutrition, fluid restriction	Limiting fluid intake to 2 L/day for patients with fluid retention or congestion that is not effectively managed with diuretics.	Weak /Low	GRADE standards	Canadian HF (2017)
Daily weight management	Keep track of the weight of patients who have heart failure and experience fluid retention or congestion that cannot be easily managed with diuretics, or patients who have notable kidney problems.	Weak /Low	GRADE standards	Canadian HF (2017)
Physical activity/Exercise training	Recommend exercise training or regular PA for HF patients who are capable of participating. Form: NR Duration/Frequency: NR	I/A	ACC/AHA Clinical Practice Guideline Recommendation Classification System (Updated May 2019)	AHA/ACC/HFSA HF (2022)
Physical activity/Exercise training	Recommend exercise for all patients who are capable of participating. Form: NR Duration/Frequency: NR	I/A	ESC Guidelines Classification Scheme	ESC HF (2021)
Physical activity/Exercise training	Frequently engaging in moderate-intensity continuous exercise can be beneficial for patients diagnosed with stable chronic HF, especially for those with reduced left ventricular ejection fraction (LVEF) Form: Moderate intensity Duration/Frequency: NR	Strong/High	GRADE methodology	Australia HF (2018)
Physical activity/Exercise training	Regular aerobic exercises for NYHA I – III patients. Form: Walking, treadmill, stationary bicycle as well as swimming Duration/Frequency:30 min/session, 5 days/week	I/B	ACC/AHA Clinical Practice Guideline Recommendation Classification System and ESC Guidelines Classification Scheme	Malaysian HF (2019)
Physical activity/Exercise training	Recommend regular exercise for all HF patients.	Strong/Moderate	GRADE standards	Canadian HF (2017)
Physical activity/Exercise training	Recommend regular exercise for HF patients to decrease hospital admissions	Strong/Moderate	GRADE standards	Canadian HF (2017)
Physical activity/Exercise training	Patients with heart failure with reduced ejection fraction (HFrEF) To enhance the quality of life, exercise therapy is utilized to lower the risk of cardiac incidents and increase overall life expectancy.	IIa/B, B/II	COR and LOE similar to those used in ACC/AHA guidelines and the ECS guidelines, and MINDS	JCS /JHFS HF (2017)
Physical activity/Exercise training	Patients with heart failure with preserved ejection fraction (HFpEF) withlow exercise capacity, utilizing exercise therapy to enhance exercise capacity.	IIa/C, B/IVa	COR and LOE similar to those used in ACC/AHA guidelines and the ECS guidelines, and MINDS	JCS /JHFS HF (2017)
Physical activity/Exercise training	Patients with heart failure after implantable cardioverter-defibrillator (ICD) or cardiac resynchronization therapy–defibrillator (CRT-D) implantation, exercise therapy to enhance exercise capacity and quality of life.	IIa/C, B/Iva	COR and LOE similar to those used in ACC/AHA guidelines and the ECS guidelines, and MINDS	JCS /JHFS HF (2017)
Physical activity/Exercise training	Resistance training can enhance the daily living activities and quality of life for individuals facing advanced deconditioning or reduced physical function. This is achieved by boosting muscle strength and endurance through the training.	IIa/C, B/IVb	COR and LOE similar to those used in ACC/AHA guidelines and the ECS guidelines, and MINDS	JCS /JHFS HF (2017)
Physical activity/Exercise training	Enhancing overall daily PA is a vital aspect of providing care for patients with stable heart failure.	I/A	LOE and GOR utilized by previously published physical therapy CPGs, but reference not given	PT HF (2020)
Physical activity/Exercise training	Aerobic exercise training for patients with stable HF, classified as NYHA Class II-III HFrEF Form: treadmill or cycle ergometer or dancing. Duration/Frequency: 20–60 min/time, 3–5 time/week for at least 8–12 weeks Intensity: 50%–90% of peak VO2 or peak work	I/A	LOE and GOR utilized by previously published physical therapy CPGs, but reference not given	PT HF (2020)
Physical activity/Exercise training	High-intensity interval exercise training in patients with stable HF, classified as NYHA Class II-III HFrE Form: Treadmill or cycle ergometer Duration/Frequency: > 35 min/time, 2–3 times/week for at least 8–12 weeks Intensity: 90%–95% of peak VO2 or peak work, HIIT total exercise doses for each week should be at least 460 kcal, 114 min, or 5.4 MET-hrs	I/A	LOE and GOR utilized by previously published physical therapy CPGs, but reference not given	PT HF (2020)
Physical activity/Exercise training	Resistance training workouts aimed at targeting the major muscle groups in both the upper and lower body are recommended for individuals with stable HF, classified as NYHA Class II-III HFrE Form: Resistance training Duration/Frequency: the whole duration for at least 8–12 weeks. 3 times/week, 45-60 min/session, 2–3 sets per muscle group Intensity:60%–80% 1RM	I/A	LOE and GOR utilized by previously published physical therapy CPGs, but reference not given	PT HF (2020)
Physical activity/Exercise training	Combined resistance and aerobic training for patients with stable HF, classified as NYHA Class II-III HFrEF Form: Combined resistance and aerobic training Duration/Frequency: Integrate 20 to 30 min of cardiovascular exercise with an equal duration of strength training, performing 2 to 3 sets for each major muscle group, three times a week, continuously for a minimum of 8 to 12 weeks Intensity: 60%–80% 1RM	II/B	LOE and GOR utilized by previously published physical therapy CPGs, but reference not given	PT HF (2020)
Physical activity/Exercise training	In outpatient settings, for stable patients with Class II and III HFrEF, whether they have baseline inspiratory muscle weakness or not, inspiratory muscle training can be conducted using threshold devices or similar tools. These devices do not rely on flow-dependent resistance and can be administered both at home and in a clinical setting Form: Inspiratory muscle training Duration/Frequency: Performing exercises for 30 min per day, with an intensity exceeding 30% of your maximal inspiratory pressure (PIMax or MIP), for 5–7 days a week, and continuing this routine for a minimum of 8–12 weeks Intensity: Greater than 30% of PIMax or MIP	I/A	LOE and GOR utilized by previously published physical therapy CPGs, but reference not given	PT HF (2020)
Physical activity/Exercise training	Outpatients diagnosed with stable HF, classified as Class II and IIIHFrEF, with or without baseline inspiratory muscle weakness, can benefit from a combination of inspiratory muscle training and aerobic exercise training. The program involves the use of a threshold device(or similar) that provides resistance but not dependent on airflow. This training regimen can be administered both at home and in a clinic setting. Form: Simultaneous training of inspiratory muscles and aerobic exercise using a threshold device or similar Duration/Frequency:30 min/day, 5–7 days/week, for at least 8–12 weeks Intensity: > 30% PIMax or MIP	II/B	LOE and GOR utilized by previously published physical therapy CPGs, but reference not given	PT HF (2020)
Smoking cessation	Smoking cessation	I/C, B/Ivb	COR and LOE similar to those used in ACC/AHA guidelines and the ECS guidelines, and MINDS	JCS /JHFS HF (2017)
Sleep Disorders	Weight loss is encouraged for OSA patients.	I/C	ACC/AHA Clinical Practice Guideline Recommendation Classification System and ESC Guidelines Classification Scheme	Malaysian HF (2019)
Sleep Disorders	CPAP improves daytime sleepiness for obstructive sleep apnea (OSA) patients.	IIa/B	ACC/AHA Clinical Practice Guideline Recommendation Classification System and ESC Guidelines Classification Scheme	Malaysian HF (2019)
Self-management	Recommend self-management to reduce the risk of hospitalization and mortality associated with HF.	I/A	ESC Guidelines Classification Scheme	ESC HF (2021)

HF, heart failure; PA, physical activity; NR, not reported;COR, Class of Recommendation; LOE, Level of Evidence; NYHA, New York Heart Association; HFrEF, Heart failure with reduced ejection fraction;1RM, 1 Repetition Maximum; OSA, obstructive sleep apnea*, CPAP,* continuous positive airway pressure

#### Prevention of heart failure

Five CPGs recommended self-managed non-pharmacological interventions to reduce the risk of developing HF [22, 44–47]. These were mainly healthy lifestyle such as regular physical activity, maintaining ideal body weight, weight reduction for overweight or obese patients, alcoholic control, and smoking cessation. There were no details recommended, except one guideline suggested physical activity with moderate intensity for more than 150 min/week [44].

#### Management of heart failure

There were seven CPGs included for the management of heart failure, the recommendations were mainly about physical activity, some include diet as well. Only one CPG suggested patients with HF and obstructive sleep apnoea to lose weight and use CPAP to improve daytime sleepiness with low to moderate LOE (B, C) [44]. There was only one CPG which suggested smoking cessation for the management of HF, but the evidence level was low (C, IVb) [45].

#### Physical activity

Physical activity/exercise were recommended by all seven included CPGs for the management of heart failure with a moderate to high degree. Three CPGs strongly recommended regular exercise/physical activity to improve functional status, symptoms, quality of life and reduce HF hospitalization, but did not give any suggestions regarding exercise form, frequency, or duration [22, 42, 47]. Another guideline suggested continuous exercise with up to moderate intensity, but no frequency or duration mentioned [46]. One CPG suggested regular aerobic exercises for New York Heart Association (NYHA) classification I-III patients, with form (e.g., walking and swimming), duration (30 min/session), and frequency (5 days/week) [44]. One CPG recommended exercise therapy for patients with different HF stages, advising resistance training for patients with advanced deconditioning and reduced physical function, with low to moderated LOE (B/II, C/IVa, C/IVb) [45]. There was one CPG developed for physical therapist by American Physical Association and was the only guideline which recommended different type of exercise with details of form, duration, and frequency for patients in different stages of HF [43].

#### Diet

Four CPGs gave some recommendations on diet for the management of HF, which were mainly about salt and fluid restriction [22, 44, 45, 47]. Four CPGs gave different recommendations regarding salt intake, the evidence level was low for three CPGs, except one guideline referenced moderate LOE(B) [44]. One CPG recommended low salt diet with less than 6 g/day [45], another CPG suggested salt intake 2-3 g/d [47], while the other two CPGs did not give the certain amount, one used “avoid adding salt” [44] and the other one used “avoid excessive sodium intake” for stage C HF [22].

The recommendations of fluid restriction were not consistent either, but LOE for all three CPGs were low, the COR was low to moderate. Two CPGs gave recommendations about fluid restriction, which were inconsistent (2L/day vs < 1.5L/day) and LOE were low [44, 47]. One guideline made weak suggestions to monitor daily weight for HF patients with fluid retention or significant renal dysfunction or congestions not easily controlled by diuretics with low quality evidence [47]. Except fluid restriction and salt, only one guideline gave suggestions about diet itself, recommending good balanced diet and avoiding adding flavouring sauces to a moderate degree (IIa) with moderate LOE (B) [44]. Alcohol control was suggested by only one guideline with low LOE(C/V1), no details provided [45].

### Atrial fibrillation

Two CPGs were included for the management of atrial fibrillation (AF), the self-managed non-pharmacological interventions were list in Table 8 [48, 49]. Weight loss together with other risk factors modification for patents with AF was suggested by both CPGs with moderate LOE (Level B). Neither of the two CPGs mentioned the details of weight loss, strategies, and goals. Physical activity was recommended to help prevent AF recurrence, the form, duration, and frequency not reported, but mentioned to avoid excessive endurance exercise by one CPG [48]. The other CPG did not report any other non-pharmacological interventions except weight loss [49].

**Table 8:** Non-pharmacological interventions recommended by the included CPGs for AF

Intervention types	Recommended self-managed non-pharmacological interventions	COR/LOE	Grading system used	Guidelines
Physical activity	Physical activity, excluding excessive endurance exercise, may promote AF. Form: NR Duration/Frequency: NR	IIa/C	ESC Guidelines Classification Scheme	ESC AF (2020)
Weight management	Weight loss, combined with risk factor modification for overweight and obese patients with AF	I/B-R	ACC/AHA Clinical Practice Guideline Recommendation Classification System (Updated August 2015)	AHA/ACC/HRS AF (2019)
Weight management	Weight loss in conjunction with the management of other risk factors for obese patients with AF	IIa/B	ESC Guidelines Classification Scheme	ESC AF (2020)
Alcohol limit	Avoid excessive alcohol	IIa/B	ESC Guidelines Classification Scheme	ESC AF (2020)

AF, atrial fibrillation; NR, not reported; COR, Class of Recommendation; LOE Level of Evidence

### Stroke

Three CPGs for the management of stroke included, and the recommendations focused on physical activity, smoking, diet, alcohol consumption, weight management [50–52] (Table 9).

**Table 9:** Non-pharmacological interventions recommended by the included CPGs for Stroke

Intervention types	Recommended self-managed non-pharmacological interventions	COR/LOE	Grading system used	Guidelines
Physical Activity	For patients experiencing stroke or TIA and are physically able, it is recommended to participate in aerobic exercises regularly. For example, engaging in moderate-intensity aerobic activity four times a week, with each session lasting at least 10 min; vigorous-intensity aerobic activity twice a week, with each session lasting at least 20 min. Form: Moderate / vigorous-intensity aerobic activity Duration/Frequency: Moderate > 10 min, 4 times/week; vigorous > 20 min, twice/week	1/C-LD	ACC/AHA Clinical Practice Guideline Recommendation Classification System (Updated May 2019)	AHA/ASA Stroke (2021)
Physical Activity	Content: To improve their cardiovascular health, individuals can enhance their sedentary routines by incorporating short breaks, such as standing or engaging in light exercise for as little as 3 min every half an hour. Form: standing or light exercise Duration/Frequency: NR	2b/B-NR	ACC/AHA Clinical Practice Guideline Recommendation Classification System (Updated May 2019)	AHA/ASA Stroke (2021)
Physical activity	Minimize inactive habits and time spent being sedentary, while progressively striving for higher activity levels that are manageable and achievable. Form: NR Duration/Frequency: NR	Evidence Level B	CSBPR writing group assigned level of evidence, GOR included in the LOE	CSBPR (2020)
Physical activity	Regular exercise program. Form: Incorporate aerobic exercise regular daily living activities. Duration/Frequency: Engage in sessions lasting 10 min each, aiming for 4 to 7 sessions per week, to reach the activity time a minimum of 150 min/week	Evidence Level B	CSBPR writing group assigned level of evidence, GOR included in the LOE	CSBPR (2020)
Physical activity	Form: Practice specific tasks to improve function in the upper and lower extremities, as well as improve gait, posture, and other daily living activities. Duration/Frequency: NR	Strong/Moderate	GRADE methodology	VA/DoD Stroke Rehab (2019)
Physical activity	Form: Engaging in cardiovascular exercises can help enhance the maximum walking speed following a stroke. Duration/Frequency: NR	Strong/Moderate	GRADE methodology	VA/DoD Stroke Rehab (2019)
Physical activity	Incorporating rhythmic auditory cueing into multimodal interventions can enhance walking speed Duration/Frequency: NR	Weak/Low	GRADE methodology	VA/DoD Stroke Rehab (2019)
Physical activity	Current evidence is inconclusive to make a recommendation for or against the use of mirror therapy for improving limb function.	Neither for nor against /Low	GRADE methodology	VA/DoD Stroke Rehab (2019)
Smoking cessation	Begins with a decrease in smoking and advances toward complete cessation.	Evidence Level B	CSBPR writing group assigned level of evidence, GOR included in the LOE	CSBPR (2020)
Smoking cessation	Avoid passive smoking	Evidence Level B	CSBPR writing group assigned level of evidence, GOR included in the LOE	CSBPR (2020)
Smoking Cessation	Stop smoking or reduce their daily smoking	1/B-NR	ACC/AHA Clinical Practice Guideline Recommendation Classification System (Updated May 2019)	AHA/ASA Stroke (2021)
Smoking Cessation	Avoid passive smoking	1/B-NR	ACC/AHA Clinical Practice Guideline Recommendation Classification System (Updated May 2019)	AHA/ASA Stroke (2021)
Diet	Follow Mediterranean-type diet	2a/B-R	ACC/AHA Clinical Practice Guideline Recommendation Classification System (Updated May 2019)	AHA/ASA Stroke (2021)
Diet	Retrict daily sodium intake by at least 1 g (salt 2.5 g)	2a/B-R	ACC/AHA Clinical Practice Guideline Recommendation Classification System (Updated May 2019)	AHA/ASA Stroke (2021)
Diet	Follow Mediterranean-type or DASH (Dietary Approach to Stop Hypertension) diet	Evidence Level B	CSBPR writing group assigned level of evidence, GOR included in the LOE	CSBPR (2020)
Diet	The objective is to limit the daily consumption of sodium to a maximum of 2000 mg (equivalent to table salt 5 g or sodium 87 mmol)	Evidence Level A	CSBPR writing group assigned level of evidence, GOR included in the LOE	CSBPR (2020)
Alcohol consumption	Individuals who consume two alcoholic beverages daily (for men) or more than one alcoholic drink daily (for women) should consider reducing or discontinuing their alcohol intake.	1/B-NR	ACC/AHA Clinical Practice Guideline Recommendation Classification System (Updated May 2019)	AHA/ASA Stroke (2021)
Alcohol consumption	Avoid heavy alcohol use, to follow Canada's Low-Risk Alcohol Drinking Guidelines (2018).		CSBPR writing group assigned level of evidence, GOR included in the LOE	CSBPR (2020)
Weight management	Achieve and maintain a waist circumference of less than 88 cm for women and 102 cm for men or maintain a BMI with the range of 18.5 to 24.9 kg/m^2^	Evidence Level B	CSBPR writing group assigned level of evidence, GOR included in the LOE	CSBPR (2020)
Weight management	Overweight patients to establish realistic and healthy weight loss objectives and develop personalized plans to attain goals.	Evidence Level B	CSBPR writing group assigned level of evidence, GOR included in the LOE	CSBPR (2020)
Mental health therapy	Using exercise as supplementary treatment for post-stroke patients withdepression or anxiety.	Weak/very low	GRADE methodology	VA/DoD Stroke Rehab (2019)
Mental health therapy	Use adjunctive therapy mind–body activities (e.g., tai chi, yoga, qigong) for post-stroke patients with depression or anxiety.	Weak/very low	GRADE methodology	VA/DoD Stroke Rehab (2019)

NR, not reported; COR, Class of Recommendation; LOE, Level of Evidence

#### Physical activity

All three CPGs gave some recommendations about physical activity with low to moderate LOE. For patients with sedentary lifestyle, one CPG encouraged patients to reduce sedentary behaviours and time, and to increase daily activity [51], the other one suggested to break up sedentary time with 30 min interval, stand or do light exercise for 3 min in between [50]. For patients who are stable and can perform physical activity, aerobic exercise was recommended by two CPGs, for example, moderate intensity 10 min, four times/week or vigorous intensity 20 min twice/week [50], regular exercise more than 10 min each time, 4 to 7 days/week to accumulate 150 min/week [51].

Another CPG strongly recommended cardiovascular exercise (e.g. walking, aquatics and rowing) to improve walking speed [52]. This guideline recommended some task-specific practice exercise such as balance activities in a standing position to improve limbs motor function, posture and daily activities as well with moderate LOE [52]. The use of rhythmic auditory cueing was recommended to improve walking speed post stroke, but the LOE was low, the ROE was weak [52]. With low quality of evidence, the guideline neither for nor against mirror therapy for post stroke patients to improve limb function [52].

#### Smoking cessation

Two CPGs recommended patients to stop smoking and avoid environmental (passive) smoke with moderate LOE [50, 51].

#### Diet

Two CPGs recommended healthy diet for patients with stroke or transient ischemic attack (TIA) [50, 51]. Both CPGs suggested Mediterranean-type diet, one CPG gave Dietary Approach to Stop Hypertension (DASH) diet as another option, both emphasized plant-based diet, encouraged fish, nuts and olive oil consumption. There was a bit inconsistency regarding sodium intake, one guideline suggested to reduce sodium intake by at least 1 g/d sodium (2.5 g/d salt) with moderate LOE [50], while the other guideline set the goal of sodium intake as no more than 2000 mg/day (5 g salt) with strong evidence (Level A) [51].

#### Alcohol consumption

One CPG advocated patients with TIA or stroke should avoid heavy alcohol use and set clear goals as per Canada’s Low-Risk Alcohol Drinking Guidelines (2018) [51]. Another CPG suggested to eliminate or reduce alcohol consumption for TIA or ischemic stroke patients who have more than two alcoholic drinks/day for men, or more than one alcoholic drink/day for women [50].

#### Weight management

Only one guideline provided recommendations regarding weight management with moderate evidence, overweight patients to set healthy weight loss goals and develop individualized plan to achieve goals, the goals are body mass index (BMI) 18.5 to 24.9 kg/m^2^, or maintain waist circumference less than 88 cm for women, less than 102 cm for men [51]. However, there was no detailed interventions/strategies about how to manage/reduce weight been provided.

#### Mental health interventions

Only one CPG suggested exercise and mind–body exercise such as taichi, yoga and qigong for mental health management, because they can be used as conjunctive treatment for patients with depression or anxiety post stroke, but with weak recommendation as the LOE very low [52].

### Peripheral artery disease

There were three CPGs about peripheral artery diseases (PADs) management included, the recommendations were mainly about smoking cessation and physical activity [31, 53, 54] (Table 10). One of the three CPGs recommended healthy diet but with low level of evidence (Level C) [54], while the other guidelines suggested weight reduction as lifestyle changes for overweight patients [53, 54].

**Table 10:** Non-pharmacological interventions recommended by the included CPGs for PAD

Intervention types	Recommended self-managed non-pharmacological interventions	COR/LOE	Grading system used	Guidelines
Smoking Cessation	Quit smoking cigarettes and other forms of tobacco	I/A	ACC/AHA Clinical Practice Guideline Recommendation Classification System (Updated August 2015)	AHA/ACC PAD (2016)
Smoking Cessation	Minimize contact with secondhand smoke in the environment	I/B-NR	ACC/AHA Clinical Practice Guideline Recommendation Classification System (Updated August 2015)	AHA/ACC PAD (2016)
Smoking Cessation	Smoking cessation	I/B	ESC Guidelines Classification Scheme	ESC PAD (2017)
Smoking Cessation	Quitting smoking and refraining from using other smoke-inhaled substances, e.g., cannabis	I/A	Systematology on the recommendations of the European Society of Cardiology and the European Society for Vascular Surgery	ESVM PAD (2019)
Smoking Cessation	E-cigarettes may be considered as an aid for smoking cessation.	IIa/C	Systematology on the recommendations of the European Society of Cardiology and the European Society for Vascular Surgery	ESVM PAD (2019)
Physical activity	Structured home-based or community- program with behavioral change techniques. Form: NR Duration/Frequency: NR	IIa/A	ACC/AHA Clinical Practice Guideline Recommendation Classification System (Updated August 2015)	AHA/ACC PAD (2016)
Physical activity	For patients with claudication Form: alternative approaches of exercise programs, such as cycling, and pain-free or low-intensity walking Duration/Frequency: NR	IIa/A	ACC/AHA Clinical Practice Guideline Recommendation Classification System (Updated August 2015)	AHA/ACC PAD (2016)
Physical activity	Physical activity Form: NR Duration/Frequency: NR	I/C	ESC Guidelines Classification Scheme	ESC PAD (2017)
Physical activity	Regular physical activity Form: NR Duration/Frequency: NR	I/C	Systematology on the recommendations of the European Society of Cardiologyand the European Society for Vascular Surgery	ESVM PAD (2019)
Diet	Healthy diet	I/C	ESC Guidelines Classification Scheme	ESC PAD (2017)
Weight management	Weight reduction in overweight patients	I/C	systematology on the recommendations of the European Society of Cardiology and the European Society for Vascular Surgery	ESVM PAD (2019)

NR, not reported; COR, Class of Recommendation; LOE, Level of Evidence

#### Smoking cessation

All three CPGs highly recommended smoking cessation for patients with PADs, with the moderate to high evidence [31, 53, 54]. Only one CPG suggested that passive smoke exposure should be avoided as it has been found associated with the development of PADs [31].

#### Physical activity

Physical activity was recommended by all three CPGs, in which one CPG supported with high LOE (Level A) [31], while the other two with low LOE (Level C) [53, 54]. Only one CPG recommended structured community- or home-based program with behavioural change to improve patients’ walking ability and functional status based on strong evidence including multiple RCTs [31]. As supported with strong evidence, this CPG also suggested upper-body ergometry, cycling, and pain-free or low-intensity walking as alternative exercise therapy for patients with claudication to improve their walking ability and functional status [31]. The other two CPGs did not give any detailed suggestion of physical activity, one CPG recommended physical activity for all patients with PADs [54], while the other one only suggested regular physical activity for PAD patients with obesity and/or physical inactivity [53].

### Hypertrophic cardiomyopathy

There was only one CPG included for the management of patients with hypertrophic cardiomyopathy (HCM), the recommendations listed in Table 11 [55]. This guideline strongly recommended mild to moderate intensity recreational exercise for most patients with HCM with moderate LOE(B-NR) [55]. The form, frequency and duration of the recreational exercise were not detailed. Based on expert opinion, it stated that most patients with HCM were reasonable to participate in low-intensity competitive sports, the recommendation level was moderate (Class 2a) [55]. Another moderate class of recommendation is for HCM patients with genotype-positive, phenotype -negative to join any competitive athletics regardless of the intensity which were supported by low LOE with limited data [55]. Overweight or obese HCM patients were strongly recommended to achieve and maintain weight loss via lifestyle interventions, but no details of lifestyle interventions provided. Instead, this guideline recommended to reduce the risk of cardiovascular events via adhering to ACC/AHA guideline on the primary prevention of cardiovascular disease [55, 56].

**Table 11:** Non-pharmacological interventions recommended by the included CPGs for HCM

Intervention types	Recommended self-managed non-pharmacological interventions	COR/LOE	Grading system used	Guidelines
Physical activity	For most patients with HCM Form: mild- to moderate-intensity recreational exercise Duration/Frequency: NR	I/B-NR	ACC/AHA Clinical Practice Guideline Recommendation Classification System (Updated May 2019)	AHA/ACC HCM (2020)
Physical activity	For most patients with HCM Form: low-intensity competitive sports Duration/Frequency: NR	2a/C-EO	ACC/AHA Clinical Practice Guideline Recommendation Classification System (Updated May 2019)	AHA/ACC HCM (2020)
Physical activity	Individuals who are genotype-positive, phenotype-negative for HCM Form: competitive athletics of any intensity Duration/Frequency: NR	2a/C-LD	ACC/AHA Clinical Practice Guideline Recommendation Classification System (Updated May 2019)	AHA/ACC HCM (2020)

NR, not reported; COR, Class of Recommendation; LOE, Level of Evidence

## Discussion

This review used AGREEII by four researchers to appraise the quality of the included 23 CPGs and extracted and synthesised the recommendations of self-managed non-pharmacological interventions for patients with CVDs. This review is critical to synthesis the results of the quality assessment and summarise the current best practice recommendations so that healthcare professionals can guide patients to choose suitable self-managed interventions and improve the outcomes in patients with CVDs by utilising the summarised evidence.

### Quality assessment of included CPGs

The ICC for the quality assessment was 0.785, indicating a substantial level of agreement between the reviewers [39]. This is an important aspect of the quality assessment, as it demonstrates the reliability of the evaluation process [39]. The AGREE II results demonstrated that the overall quality of the included CPGs was high, with a mean overall standardized percentage of 84.9%, the average percentage of the six domains ranged from 64.9% to 97.3%. Based on the predefined standard, twenty CPGs with a mean percentage over 70% were rated as "recommended" (high quality), the remaining three guidelines [20, 45, 47] were appraised "recommended with modification" (moderate quality) due to standardized percentages between 40 and 70% in over three domains. Therefore, all twenty-three CPGs were recommended for use either with or without modifications. It is important to consider the individual domain scores for each guideline as well, as they provide insights into the specific areas where improvement is needed.

The domain of "Editorial Independence" received the highest average standardized percentage (93.47%). This suggests that the majority of the writing committees developed the CPGs without interference from funding bodies. However, one guideline had a score of 47.9% in this domain [47], indicating potential influence from the funding body [38]. The domain of "Clarity and Presentation" ranked second highest, with an average of 90.47%, suggesting that most CPGs provide clear and specific recommendations. The domains of "Scope and Purpose" and "Stakeholder Involvement" received average standardized percentages of 88.35% and 83.4%, respectively. These scores indicate that most CPGs were developed with well-defined objectives, a clear focus on the health questions and target population, and the involvement of relevant stakeholders such as healthcare professionals, patients, and experts from various disciplines [38].

Regarding the domain of “Rigour of Development”, all selected CPGs mentioned using systematic review, but seven of the selected CPGs did not use systematic methods to search nor mention clear evidence selecting criteria [17, 20, 42, 45, 48, 53, 54], five CPGs did not clearly describe how to formulate recommendations [17, 20, 42, 48, 54], which made the whole domain score low. Systematic approach should be used to search, select, and develop evidence-based recommendations for healthcare professionals. In addition, the updating procedure were not provided by about half of the selected CPGs [17, 20, 42, 45–48, 51–53, 53, 54], some provided update schedules but the update frequency varied, such as one CPG suggested to revise every 3 to 4 years [21], some suggested to revise CPG at least every 5 years [40, 41, 49] while one CPG suggested revise full guideline in about 6-year cycle [49]. As the research progresses considerably quicker than the interval of CPG update, the CPG writing committee should review new data on an ongoing basis to make sure the recommendations remain current [22, 55]. The lowest mean percentage with 75.41% of “Applicability” domain indicates that the facilitators and barriers, monitoring and/or auditing criteria of the CPG application were not clearly presented. The results of the “applicability” domain suggest that more work needed for facilitators and strategies to overcome barriers of implementing CPG, such as seeking feedback from stakeholders or pilot testing before CPG widespread implementation [38].

### Content analysis of included CPGs

The included CPGs provided some self-managed non-pharmacological interventions with COR and LOE, the interventions mainly focus on diet, weight management, physical activity, alcohol limitation and smoking cessation. Regular physical activity or exercise is the only core self-managed intervention strongly recommended for patients with CVDs by most included CPGs except one CPG [49]. Physical activity or exercise can help improve exercise capacity, function status, quality of life and reduce hospitalisation supported in patients with CVDs with moderate to high level of evidence [22, 42, 44, 47].To reduce sedentary behaviours and time, and increase physical activity levels gradually are agreed recommendations of physical activity [17, 18, 43, 51, 51].The agreed form and duration are at least 150 min of moderate intensity exercise (e.g. fast walking, stationary bicycle, slow to moderate swimming) or 75 min of intense/vigorous exercise (e.g. weight training, jog/run > 8 km/hr, stair-treadmill) or a combination of both for all age patients who are medically stable [17, 19, 20, 40, 41, 51]. But some CPGs did not provide physical activity details, such as the form and duration [45, 47, 48, 54].

Healthy balanced diet was recommended for patients with CVDs by most included CPGs. The dietary pattern and food groups were explained clearly by CPGs for CVDs and one CPG for cardiac rehabilitation [17–21], while other CPGs did not provide details regarding dietary details such as food groups. The recommended dietary pattern is Mediterranean or similar diet, with more plant, less animal based food, encouraged to consume whole grains, fruits, vegetables, nuts and fish, with strong COR and moderate to high LOE [17–19, 21]. Added sugar was suggested to limit to less than 10% of total energy intake, and daily salt intake less than 5 g was recommended by three CPGs with strong COR and high LOE [17, 19, 21]. Two CPGs recommended less than 6 g salt intake with weak COR and low LOE [18, 45]. while one CPG recommended HF patients to restrict salt intake to 2-3 g/d with weak recommendation based on low quality evidence [47]. There is also some inconsistency in omega-3 supplementation, only one CPG recommended omega-3 as the secondary prevention of CVDs with moderate COR and moderate LOE [20], while other CPGs not recommended [18, 19].

Abstinence or reduction of alcohol consumption were recommended, but no self-managed strategies recommended by included CPGs. The limit of maximum alcohol consumption was inconsistent, one CPG strongly recommended less than 100 g/week, the other CPG moderately suggest less than 1 drink or 10 g/day for non-pregnant women, less than 2 drink or 20 g/day for men, both CPG supported with moderate level of evidence, while another CPG moderately recommended desirable to avoid, limit to 2 shots/day for men(20 mg) and 1 shot/day for women (10 mg) based on moderate LOE [21]. Other CPGs only recommended the restriction of alcohol with low to moderate LOE, but did not provided details such as the maximum alcohol consumption [45, 48, 50, 51].

There were also some ambiguities in the recommendation for the goal of weight loss as well, two CPGs strongly recommended 5–10% weight loss and maintain 1–2 year before attempting more weight loss with moderate LOE [19, 41], one CPG highly recommended at least 3 kg and maintain this reduction with moderate LOE [18]. Regarding the waist circumstances, one CPG recommended the goal for patients with TIA or stroke is to achieve and maintain waist circumstances less than 102 cm for men, 88 cm for women, or BMI from 18.5 to 24.9 with moderate LOE [51], another CPG for CVD prevention strongly recommended less than 90 cm for men and 80 cm for women with high quality LOE [19]. Some CPGs strongly recommended self-managed strategies of weight management with moderate LOE, such as calories restrictions [19, 20, 41]increased physical activity and behavioural modifications [19].while other CPGs did not provide the goal or self-managed strategies of weight management [40, 46, 48, 49].

Smoking cessation were strongly recommended with moderate to high LOE by all included CPGs except the CPG for HCM, but the details of self-managed smoking cessation interventions were missing by most CPGs. Exercise, tai chi, qigong, and yoga were recommended as possible self-management strategies for mental health therapy in post stroke patients with depression or anxiety symptoms by one CPG, but the recommendation level was low with very low level of evidence [52].

### Implications for clinical practice and future research

#### Implications for clinical practice

The results of this systematic review can be used to inform decision-making processes in clinical practice, ensuring that the self-managed non-pharmacological interventions healthcare professionals recommend to patients are based on the best available evidence. The recommendations extracted from the included CPGs did not specify any particular age group,  indicating a more  generalised approach to self-managed non-pharmacological interventions for CVDs rather than age-specific interventions. The successful implementation of self-managed non-pharmacological interventions in clinical settings requires a strong partnership among healthcare professionals, including general practitioners, nurses, dietitians, and physiotherapists. Additionally, the findings of this systematic review can help shape the educational programs for healthcare professionals to promote the effective implementation of self-managed non-pharmacological interventions for CVDs. Healthcare organizations (e.g., hospitals and community healthcare centers) can also utilize  the findings to identify areas for improvement, develop targeted interventions, and monitor the impact of these interventions on cardiovascular  outcomes.

The methodological quality appraisal results from  this systematic review can assist guideline developers in improving  the quality of updated guidelines in the future, especially in domains such as "Rigor of development" and "Applicability." The "Rigor of development" domain should be improved with systematic reviews of evidence, and the "Applicability" domain needs to be strengthened by considering facilitators and barriers of guideline  application, along with monitoring and/or auditing criteria.

#### Implications for future research and policy

The results from this systematic review will inform the development of the self-managed non-pharmacological intervention protocols and provide a foundation for further investigation in this field. Researchers can explore areas where inconsistencies or uncertainties exist among the recommendations and supported evidence, such as refining specific recommendations with limited evidence or examining the ideal goals of weight reduction for patients with CVDs. Large-scale, high-quality randomized controlled trials are needed to provide more robust evidence for self-managed non-pharmacological interventions, particularly for those with limited supporting evidence. In future research, more intricate quantitative analyses can be incorporated into the quality appraisal process by using AGREE II. For example, linear regression modelling can be used to explore associations between the quality assessment findings, also can help investigate potential correlations with high quality versus low quality CPGs.

Policy makers can also benefit from the results  of this systematic review, as they can help inform the development of evidence-based policies and self-managed non-pharmacological programs that promote the prevention and management of CVDs. By considering the results of this review, policy makers can design strategies that effectively support the implementation of self-managed non-pharmacological interventions in various healthcare settings, ultimately contributing to improved patient outcomes and public health.

### Study limitations

This systematic review has several limitations. Firstly,  only CPGs published in English were included in this systematic review, guidelines published in other languages were not included. Secondly, the content analysis focused primarily on summarizing the recommendations of self-managed non-pharmacological interventions for CVDs, which may not capture the full scope of recommendations within the included guidelines. This limitation could result in a narrow overview of the guidelines' content, potentially omitting relevant information that could be valuable for healthcare professionals and researchers, such as other non-pharmacological recommendations that cannot be self-managed and require a multidisciplinary team approach. Lastly, the subjective assessment of the included CPGs by four reviewers could be considered a limitation, but the average ICC of 0.785 indicates good reliability, suggesting that the assessment process was consistent and reliable among the reviewers involved in this systematic review. Furthermore, database searches were performed in May 2022 to ensure the inclusion of all pertinent CPGs from the past five years. It is crucial to acknowledge that the results of this systematic review could be constrained by its termination as of May 2022. Due to the ongoing progress in  healthcare research and guidelines, newer interventions and updated guidelines may have surfaced after this date. Consequently, some results of this systematic review may not align with the most recent recommendations for self-managed non-pharmacological interventions for CVDs.

## Conclusion

In summary, the latest CPGs concerning the management of CVDs are of high quality, as demonstrated by the AGREE II results. The majority of the summarized self-managed non-pharmacological interventions were strongly recommended, supported by moderate to high-quality levels of evidence. Although some inconsistencies were observed in the summarized recommendations among the included CPGs, the key recommendations of physical activity and a healthy diet, supported by high levels of evidence, are expected to benefit patients with CVDs. Healthcare professionals and researchers can use the findings of this review to inform the design of self-managed non-pharmacological interventions for patients with CVDs.

## Data Availability

Not applicable.
